# Maturation and Activity of Sterol Regulatory Element Binding Protein 1 Is Inhibited by Acyl-CoA Binding Domain Containing 3

**DOI:** 10.1371/journal.pone.0049906

**Published:** 2012-11-14

**Authors:** Yong Chen, Vishala Patel, Sookhee Bang, Natalie Cohen, John Millar, Sangwon F. Kim

**Affiliations:** 1 Department of Psychiatry, Center for Neurobiology and Behavior, University of Pennsylvania, Philadelphia, Pennsylvania, United States of America; 2 Metabolic Tracer Resource, Institute of Diabetes, Obesity & Metabolism, University of Pennsylvania, Philadelphia, Pennsylvania, United States of America; Graduate School of Medicine, the University of Tokyo, Japan

## Abstract

Imbalance of lipid metabolism has been linked with pathogenesis of a variety of human pathological conditions such as diabetes, obesity, cancer and neurodegeneration. Sterol regulatory element binding proteins (SREBPs) are the master transcription factors controlling the homeostasis of fatty acids and cholesterol in the body. Transcription, expression, and activity of SREBPs are regulated by various nutritional, hormonal or stressful stimuli, yet the molecular and cellular mechanisms involved in these adaptative responses remains elusive. In the present study, we found that overexpressed acyl-CoA binding domain containing 3 (ACBD3), a Golgi-associated protein, dramatically inhibited SREBP1-sensitive promoter activity of fatty acid synthase (FASN). Moreover, lipid deprivation-stimulated SREBP1 maturation was significantly attenuated by ACBD3. With cell fractionation, gene knockdown and immunoprecipitation assays, it was showed that ACBD3 blocked intracellular maturation of SREBP1 probably through directly binding with the lipid regulator rather than disrupted SREBP1-SCAP-Insig1 interaction. Further investigation revealed that acyl-CoA domain-containing N-terminal sequence of ACBD3 contributed to its inhibitory effects on the production of nuclear SREBP1. In addition, mRNA and protein levels of FASN and de novo palmitate biosynthesis were remarkably reduced in cells overexpressed with ACBD3. These findings suggest that ACBD3 plays an essential role in maintaining lipid homeostasis via regulating SREBP1's processing pathway and thus impacting cellular lipogenesis.

## Introduction

Sterol regulatory element binding proteins (SREBPs) are a family of basic-helix-loop- helix leucine-zipper (bHLH-LZ) transcription factors. There are three well-characterized SREBP isoforms in mammals: SREBP1A, SREBP1C and SREBP2 [1]. They are the master regulators of lipid homeostasis in the cells through controlling expression of over 30 genes, such as fatty acid synthase (FASN), acetyl-CoA carboxylase (ACC), low-density lipoprotein receptor, HMG-CoA reductase, stearoyl-CoA desaturase, ATP citrate lysase and Glycerol-3-phosphate acyltransferase [2], [3]. This list of SREBP target genes is continuously expanded. For an example, a recent report by Walker et al. revealed that S-adenosylmethionine synthetase type 1, methylenetetrahydrofolate reductase, methionine synthase and several other related genes were regulated by SREBP1 [4]. Those SREBP target genes are required for biosynthesis of fatty acid, cholesterol, triglyceride and phospholipids. The activity of SREBP1A markedly increases the expression of genes involved in both cholesterol and fatty acid. SREBP1C demonstrates a slight selectivity, but lower activity, on fatty acid and triglyceride synthesis. SREBP2 causes a preferential induction of genes related to cholesterol biosynthesis [5], [6].

SREBPs are initially synthesized and are located in the endoplasmic reticulum (ER) as full-length precursor proteins (fSREBPs) forming a complex with SREBP cleavage-activating protein (SCAP) and insulin induced gene (Insig). Upon receiving activation signals, the fSREBPs are transported to Golgi apparatus where they are cleaved sequentially by site 1 protease and site 2 protease. The newly-generated N-terminal fragments (nSREBPs) then translocate into nucleus, bind on both E-boxes and sterol regulatory elements within the promoter regions of their targets and stimulate lipogenic gene transcription [1], [6].

Acyl-coenzyme A binding domain containing 3 (ACBD3) is recognized as a Golgi-associated protein, although small levels of protein expression can be detected in cytoplasm, ER and mitochondria [7], [8]. During cell mitosis, the Golgi-associated section of ACBD3 is released into cytosol due to Golgi structure disintegration [8]. Previous studies have suggested this ubiquitously expressed protein plays several biological functions such as lipid metabolism, iron-related neurotoxicity, membrane trafficking, neuronal division, neurogenesis, development, and virus replication. Through involving in protein kinase A-mediated signaling, ACBD3 facilitates cholesterol entry into mitochondria and thus steroidogenesis [9]. We previously showed that ACBD3 bound with Dexras1 and DMT1 to enormously promote iron uptake in neurons, which contributed to the NMDA-induced excitotoxicity [10]. Through interacting with integral Golgi membrane protein giantin, ACBD3 is implicated in the structure maintenance of Golgi and ER-to-Golgi protein trafficking [11]. During mitotic Golgi fragmentation, ACBD3 was found to physically interact with Numb protein and functionally modulate Numb signaling, which is critical for neural progenitor cell's asymmetric division and neurogenesis [12]. Embryonic lethality of ACBD3 homozygous knockout mice indicates that this protein is critical for embryonic development [12]. Two recent studies revealed that ACBD3 formed protein complexes with Phosphatidylinositol 4-kinase IIIβ and viral nonstructural proteins to regulate RNA replication of picornavirus in the host cells [13], [14].

Many fatty acid-related lipid-binding proteins such as acyl-CoA binding domain containing proteins (ACBDs), fatty acid binding proteins (FABPs) and fatty acid transport proteins (FATPs), have been strongly implicated into lipid transport, metabolism, storage and signaling, which may influence membrane molecular composition, cellular energy homeostasis, and signal transduction within cells [8], [15]–[17]. Since ACBD3 contains an acyl CoA-binding domain at its N-terminus and is involved in steroid biosynthesis, and SREBP proteins are the regulators of lipid homeostasis in cells, we were interested to investigate whether ACBD3 plays a role in homeostasis of lipids, especially fatty acids, and how ACBD3 affects expression and function of SREBP1. In the present study, we showed that overexpressed ACBD3 down-regulated the nuclear mature form of SREBP1 and inhibited its transcriptional activity, ultimately attenuating FASN expression and activity.

## Results

### ACBD3 inhibits SREBP1-promoted FASN transcription

**Figure 1:**
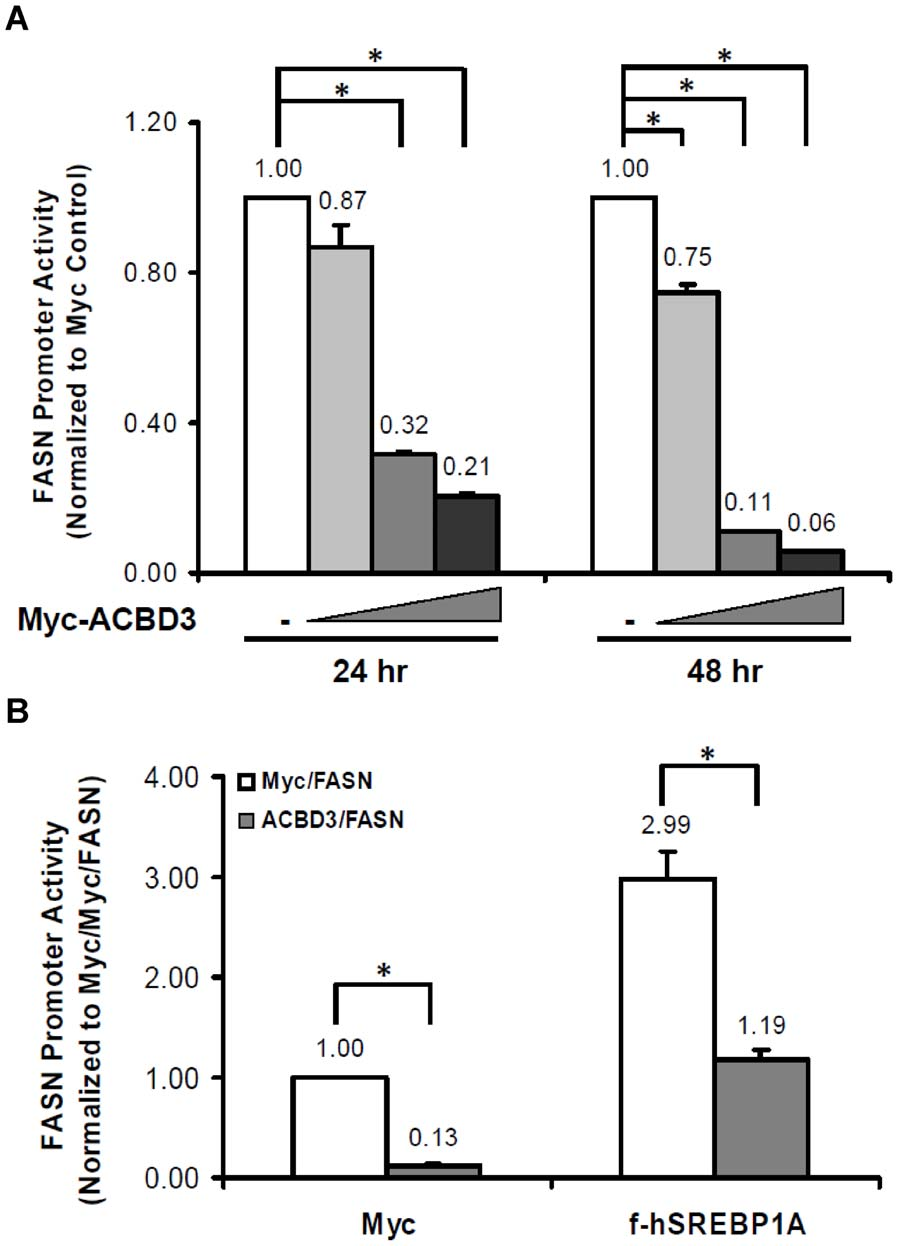
ACBD3 inhibits SREBP1-promoted *FASN* transcription. FASN promoter luciferase vector was co-transfected into HEK293T cells with Myc-ACBD3 plasmid (varied amount: 0.1–1.5 µg, (A.); or fixed 1.0 µg, (B.)) in the absence (A.) or presence (B.) of Myc-hSREBP1A (full-length) vector. Twenty-four hours (A.) or 48 hr (A. and B.) after transfection, cells were harvested for luciferase assay as described in the “Methods”. Each value represents the mean ± S.E. of triplicate experiments. *: p<0.05 as determined by one-way ANOVA followed by a Tukey post hoc test.

Since FASN is one SREBP1 target gene and an essential multi-function enzyme in the de novo fatty acid biosynthesis pathway [18], we employed a luciferase reporter vector, encoding a sterol regulatory element (SRE)-containing FASN promoter sequence fused with firefly luciferase to assess how ACBD3 overexpression changed FASN promoter activity. As shown in Figure 1A, in the presence of overexpressed Myc-ACBD3, luciferase expression was dose- and time-dependently attenuated. Forty-eight hours after transfection, FASN promoter activity was inhibited almost completely (94%). We decided to examine further whether ACBD3-mediated down-regulation of FASN promoter activity is modulated by SREBP1. We overexpressed full length SREBP1A and FASN reporter construct with or without ACBD3 and measured luciferase activity. We found that full-length SREBP1A-boosted FASN promoter activity was also significantly decreased by co-expressed ACBD3, suggesting this ACBD3-mediated inhibition of FASN promoter activity works through SREBP1 (Figure 1B).

### ACBD3 blocks lipid deprivation-stimulated SREBP1 maturation

**Figure 2:**
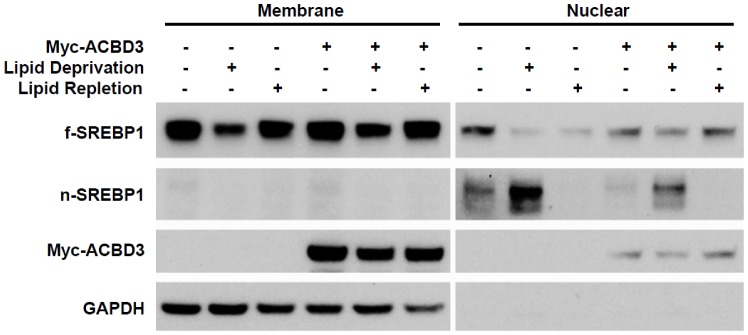
ACBD3 blocks lipid deprivation-stimulated SREBP1 maturation. HeLa cells were transfected with or without Myc-ACBD3 on Day 1. Growth medium was changed with fresh completed medium (DMEM supplemented with 10% FBS and 100 U/ml PS), lipid deprivation medium (DMEM supplemented with 10% delipidated FBS, 100 U/ml PS, 50 µM Mevastatin, 50 µM Mevalonic acid sodium) or lipid repletion medium (lipid deprivation medium supplemented with 10 µg/ml cholesterol plus 1 µg/ml 25-hydroxycholesterol) on Day 2. Twenty hours later, cells were washed and harvested followed by cellular fractionation as described in “Methods”.

Intracellular SREBP processing and maturation can be stimulated by depletion of cellular lipid [19]. We speculated that ACBD3 might function to interfere with SREBP1's negative feedback to cellular levels of lipids. Consequently, we overexpressed Myc-ACBD3 or control vectors into HeLa cells [20], [21] and examined SREBP processing. The results in Figure 2 show that in the empty-control cells growing under the lipid-depletion condition, endogenous expression of nuclear active SREBP1 was largely enhanced and correspondingly that of the membrane precursor form was significantly decreased. On the other hand, lipid-repletion incubation gave rise to the opposite response for nuclear SREBP1. Those observations suggested that our lipid manipulation successfully initiated SREBP1 adaptative responses. When ACBD3-overexpressing cells were exposed to the lipid-deprivation condition, a prominent attenuation of the depletion-initiated SREBP1 feedback was observed, even though the upregulating trend of nSREBP still existed. This indicates that ACBD3 may play a role in regulating lipid homeostasis in the cells via negatively influencing SREBP1.

### ACBD3 attenuates maturation of full-length SREBP1

**Figure 3:**
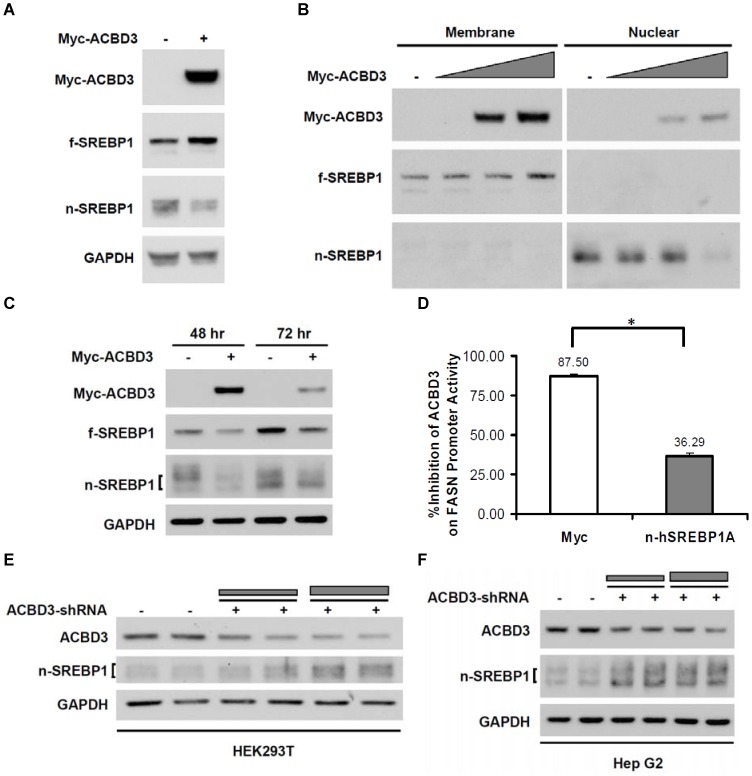
ACBD3 attenuates maturation of full-length SREBP1. HEK293T cells were transfected with fixed (A.) or different (B.) amount of Myc-ACBD3 vector. Forty hours after transfection, cells were harvested for SDS-PAGE/Western blotting analysis either directly (A.) or following cellular fractionation (B.) as described in “Methods”. Full-length and nuclear forms of SREBP1 are showed as f- and n-SREBP1 in the figure, respectively. Protein expression of SREBP1 was also evaluated in Hep G2 cells overexpressing Myc-ACBD3 for 48 or 72 hr (C.) To find out whether nuclear SREBP1 may rescue ACBD3-inhibited FASN promoter activity, FASN promoter luciferase vector was co-transfected into HEK293T cells with Myc-ACBD3 plasmid in the absence or presence of Myc-tagged nSREBP1A (1–490) vector (D.). Forty-eight hours after transfection, cells were harvested for luciferase assay as described in the “Methods”. Each value represents the mean ± S.E. of triplicate experiments. *: p<0.05 as determined by Student's *t*-test. In order to assess effect of ACBD3 gene knockdown on SREBP1 expression, control or ACBD3-shRNA vectors were transfected into HEK293T (E.) or Hep G2 (F.) cells. Cells were harvested 72 hr after transfection for SDS-PAGE/Western blotting analysis as described in “Methods”. GAPDH staining was utilized as loading control.

Since the nuclear mature forms of SREBPs are responsible for their transactivation function to stimulate lipogenic gene transcription, we investigated whether ACBD3-mediated reduction of FASN reporter activity is due to a decrease in the levels of nSREBP. We overexpressed Myc-ACBD3 in HEK293T cells and examined expression levels of both unprocessed fSREBP and N-terminal nuclear active nSREBP. We found that, in the cell total lysate, overexpressed Myc-ACBD3 greatly down-regulated mature form (∼60 KDa) but up-regulated full-length form (∼120 KDa) of endogenous SREBP1 in HEK293T cells (Figure 3A). Overexpression of GST- or GFP-tagged ACBD3 caused the identical regulation pattern of SREBP1 as the Myc-tagged protein did (data not shown). Furthermore, cell fractionation experiments (Figure 3B) showed that ACBD3 reduced the nuclear mature SREBP1 but increased the full-length membrane form in a dose-dependent manner. ACBD3's down-regulating effect on nSREBP1 was also observed in Hep G2 human hepatic cells (Figure 3C), which are considered to be more metabolism-relevant. In consistency with this decreased nSREBP1 expression, we found that overexpression of nuclear fragment (1–490) of hSREBP1A significantly rescued the ACBD3-inhibited FASN promoter activity in HEK293T cells (Figure 3D). More strikingly, after knocking down endogenous ACBD3, nuclear SREBP1 expression was enhanced in both HEK293T and Hep G2 cells (Figure 3E and 3F, respectively). All these results strongly suggest that ACBD3 intrinsically plays a negative role in SREBP1 protein maturation.

### ACBD3 binds to full-length SREBP1

**Figure 4:**
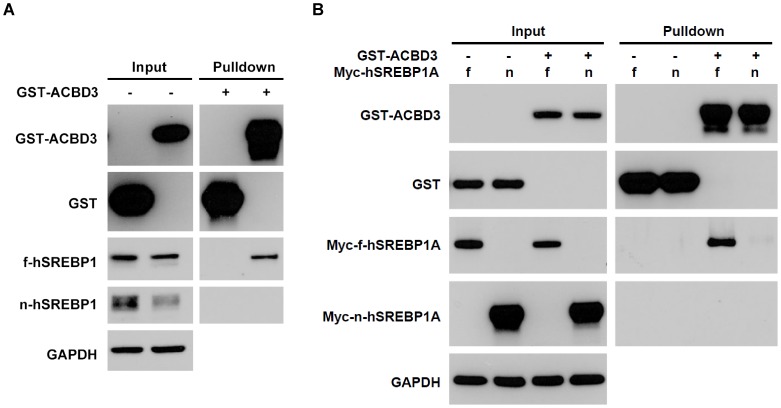
ACBD3 binds to full-length SREBP1. For studying protein-protein interaction, GST-ACBD3 in absence (A.) or presence (B.) of Myc-hSREBP1A full-length (f) form or nuclear active form (n) were expressed in the HEK293T cell. Then immunoprecipitation experiments were performed 48 hr after transfection as described in “Methods”. GAPDH staining was utilized as loading control.

Because ACBD3 is mainly a Golgi-associated protein [8] and SREBP1 also distributes on Golgi structure, we investigated whether these two proteins interacted physically. According to Figure 4A, GST-tagged ACBD3 co-immunoprecipitated with endogenous precursor fSREBP1 but not mature nSREBP1. In addition, overexpressed nuclear hSREBP1A (1–490) still could not be pulled-down by GST-ACBD3 (Figure 4B). All these observations indicate that ACBD3 physically interacts with full-length SREBP1 protein outside the cell nuclei. It is very likely that the interaction between the two proteins disrupts SREBP1 intracellular processing, resulting in the accumulation of the full-length SREBP1 on extranuclear membrane structures, and thus reducing the generation of the nuclear form.

### ACBD3 does not disturb physical SREBP1-SCAP-Insig1 interaction

It is well-known that SREBP1 forms complex with Insig1 through mutually binding to SCAP on the ER membrane at the normal condition [22], and that factors disrupting the ternary interaction (for an example, lipid deprivation) may facilitate SREBP1's ER-to-Golgi translocation followed by enhanced cleavage and maturation [1], [6]. It is plausible to speculate that ACBD3 influences SREBP1's maturation via interfering SREBP1-SCAP-Insig interaction. Hence, we first examined whether expression of SCAP and Insig1 was changed in cells overexpressing Myc-ACBD3. As shown in Figure S1, ACBD3 had no impact on the steady-state level of endogenous SCAP and Insig1 in both HEK293T and Hep G2 cells. Interestingly, knockdown of endogenous ACBD3 had no effect on the expression of these two SREBP-binding proteins, either (Figure S2). Moreover, co-immunoprecipitation assay using anti-SCAP antibody (Figure S3) clearly showed that presence of overexpressed ACBD3 did not disturb the ternary complex in the cells. Thus, ACBD3's inhibitory function on SREBP1 intracellular processing is not due to changed SREBP1-SCAP-Insig1 interaction.

### N-terminal structure of ACBD3 is important for its inhibitory effects on SREBP1

**Figure 5:**
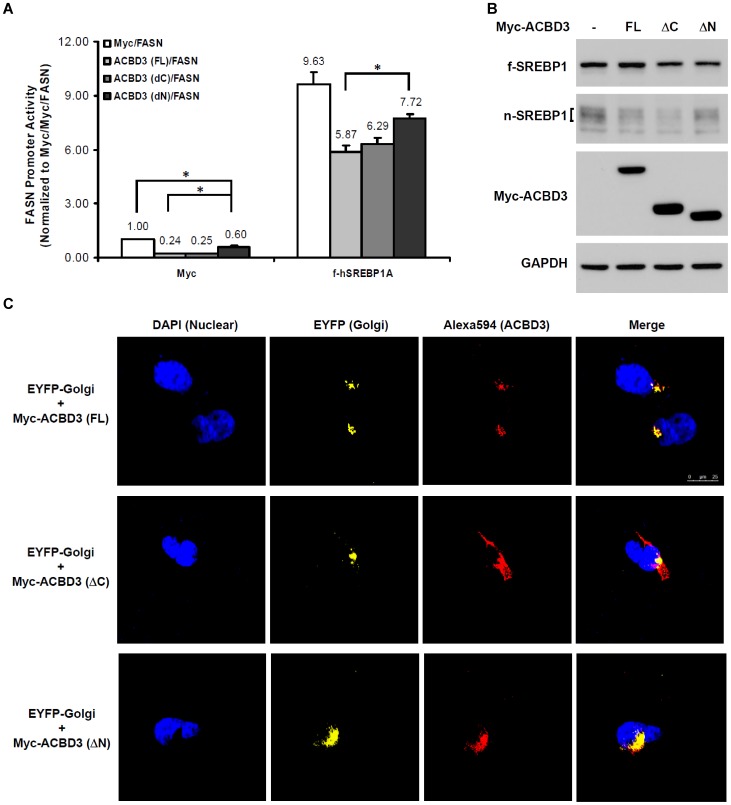
N-terminal structure of ACBD3 is important for its inhibitory effects on SREBP1. Myc-ACBD3 full-length (FL), C-terminal GOLD domain deletion mutant (ΔC) or N-terminal deletion mutant (ΔN) was co-expressed in HEK293T cells with FASN promoter luciferase in the absence or presence of Myc-hSREBP1A full-length for 48 hr (A.). Cells were then harvested for luciferase assay as described in “Methods”. Each value represents the mean ± S.E. of triplicate experiments. *: p<0.05 as determined by one-way ANOVA followed by a Tukey post hoc test. To investigate which structural part of ACBD3 protein is required for its inhibitory function, FL, ΔC and ΔN ACBD3 were overexpressed in HEK293T cells for 2 days (B.). After harvest, cells were lysed and subjected to SDS-PAGE/immunoblotting analysis as described in “Methods”. For locating ACBD3 proteins in the cells, different Myc-ACBD3 constructs were co-transfected with pEYFP-Golgi in HEK293T cells for 40 hr (C.). Immunofluorescence staining and confocal microscopic imaging were subsequently performed as described in “Methods”. Fluorophores DAPI (blue), EYFP (yellow) and Alexa fluor 594 (red) were employed to label nucleus, Golgi apparatus and Myc-tagged ACBD3, respectively. Scale bar, 25 µm.

ACBD3 contains two very distinct domains, ACB domain (80–171) and GOLD domain (Golgi Dynamics, 381–526 residues), at the N-terminal and C-terminal regions, respectively. Hence we wondered whether any of these domains specifically played a role in regulating SREBP1. With the luciferase assay, it was shown that the deletion of the N-terminal region (1–171) in ACBD3 (ΔN) significantly attenuated ACBD3-mediated suppression of FASN promoter activity, (40% vs. 76%, Figure 5A). This change pattern did not vary in the presence of overexpressed SREBP1A. Similarly, even though this mutant was found to disrupt the production of nuclear mature form of SREBP1, its disruptive effect was still smaller than that mediated by full-length ACBD3 (Figure 5B). However, the other deletion mutant of ACBD3 without the C-terminal GOLD domain (ΔC) showed comparable inhibitory effect on SREBP1's maturation and transcriptional function as the full-length protein. Our immunofluorescence confocal imaging studies revealed that most of the overexpressed full-length and ΔN ACBD3 proteins were localized on Golgi apparatus while C-terminal deletion (ΔC) largely lost this subcellular selectivity (Figure 5C). In addition, Golgi structure appeared not to be affected by overexpression of all three constructs. Taken together, these results suggest that ACB domain-containing N-terminal sequence of ACBD3 plays an important role in its regulatory effects on SREBP1.

### ACBD3 down-regulates transcription and expression of FASN and ACC and reduces de novo synthesis of palmitate

**Figure 6:**
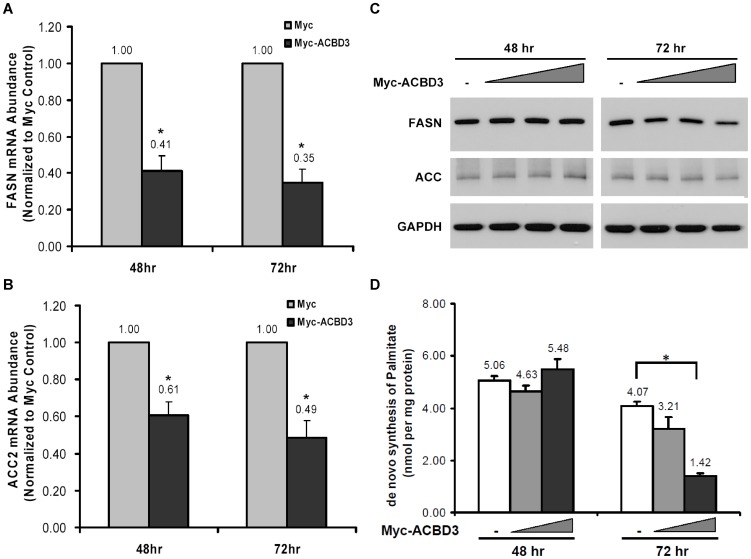
ACBD3 down-regulates transcription and expression of FASN and ACC and reduces de novo synthesis of palmitate. HEK293T cells were transfected with Myc-ACBD3 vector. Forty-eight or 72 hr after transfection, cells were harvested for RNA extraction and RT-qPCR analysis as described in “Methods” to assess the effect of ACBD3 on mRNA abundance of FASN (A.) and ACC2 (B.) genes. Each value represents the mean ± S.E. of triplicate experiments. *: p<0.05 as determined by Student's *t*-test. Similar cells were harvested and subjected to SDS-PAGE/immunoblotting analysis as described in “Methods” to study effect of ACBD3 on protein level of FASN and ACC (C.). For quantifying palmitate de novo synthesis, cell growth medium was replaced with one containing 5% D2O (diluted in the complete medium) at 24 hr or 48 hr after transfection (D.). Following 24-hours incubation, cells were harvested and lipids were extracted and determined as described in “Methods”. Each value represents the mean ± S.E. of triplicate experiments. *: p<0.05 as determined by one-way ANOVA followed by a Tukey post hoc test.

Acetyl-CoA carboxylase (ACC) and FASN are two downstream targets regulated transcriptionally by SREBP1. These two lipogenic enzymes function sequentially for synthesizing palmitate, the first fatty acid product, from acetyl-CoA in cytoplasm. By further elongation and/or desaturation on the basis of palmitate, more derivatives could be generated [23]. Results from above experiments logically leads us to the assumption that mRNA transcription and protein expression of SREBP1 downstream targets should be also suppressed by overexpression of ACBD3 following the decreased SREBP1 maturation. Indeed, overexpressed ACBD3 in HEK293T cells significantly decreased mRNA level of *FASN* and *ACC2* Figure 6A and 6B). It was further found that Myc-ACBD3 reduced nuclear mature form of endogenous SREBP1 at 48 hours after transfection even though it did not concomitantly decrease the steady-state level of both FASN and ACC (Figure 6C). On the other hand, at 72 hours after transfection, ACBD3 dose-dependently down-regulated not only nuclear SREBP1 but also FASN and ACC. Consistently, protein expression of FASN in Hep G2 cells was lowered by overexpressed ACBD3 at both 48 and 72 hours after transfection (Figure S1B).

Since we observed that ACBD3 not only reduced processing of SREBP1 but also its downstream target FASN, we investigated whether ACBD3 indeed reduces the lipid production. We determined de novo synthesis of palmitate by employing D2O labeling and Mass Spectrometry. We found that level of de novo palmitate in HEK293T cells with 48-hours ACBD3 overexpression did not show any significant change compared to that in control cells. On the contrary, if allowing overexpressed ACBD3 to be present in the cells for longer time (72 hours), we could see the reduction of palmitate synthesis (Figure 6D). The change in cells transfected with higher doses of ACBD3 was statistically significant. Moreover, this ACBD3-mediated blockade of de novo palmitate biosynthesis was similarly observed in Hep G2 cells (Figure S4).

## Discussion

In the present study, we have identified lipid-binding protein ACBD3 as a new binding partner and maturation modulator of SREBP1, and demonstrated ACBD3's ability to influence de novo biosynthesis of fatty acids by regulating FASN and ACC. These data significantly broaden our views on the biological roles played by ACBD3 and provide novel insights into the mechanisms underlying how lipid-binding proteins affect lipid homeostasis.

In cells, there are several families of lipid-binding proteins such as ACBDs, FATPs and FABPs, which function coordinately to regulate homeostasis and function of fatty acids. Entry of free fatty acids into the cells can be mediated through either passively diffusing through cell membrane or actively uptaking by FATPs. Intracellular fatty acids are then bound to either FABPs or ACBDs following conversion to fatty acyl-CoA esters by acyl-CoA synthetases [24], [25]. In those forms of complexes with chaperone proteins, fatty acids may be transported to various cellular compartments for executing their multifaceted biological functions, such as to the ER for synthesis of biological membrane and more complex lipids, to mitochondria for generating ATP via β-oxidation, to the lumen of secretory pathway for membrane protein lipidation, to the nucleus for signaling lipid-mediated transcription, or to lipid droplets for energy storage as triglycerides [16], [26], [27].

Considering their amphipathic physicochemical properties and complicated biological functions, fatty acyl-CoA esters need to be tightly regulated inside the cells. Accumulating evidence has demonstrated that the ACBD family proteins, acting as intracellular acyl-CoA pool formers and transporters, play critical roles in lipid metabolism and energy homeostasis [8]. ACBD1 (also known as Acyl-CoA Binding Protein or ACBP) is the prototypical ACBD family member and has been investigated most intensively. This 87-amino-acids protein, whose ACB domain covers its entire sequence, was found to bind with acyl-CoA ester with a *K_d_* at a nanomolar level [28]. Homozygous deficiency of this housekeeping gene resulted in embryonic lethality in mice, and siRNA-mediated ACBD1 knockdown in HepG2 cells initiated transcriptional changes on 22 genes related to cellular lipid metabolic process and caused significant reduction of palmitate content [29], [30]. Observations that augmentation of muscular fatty acyl-CoA in obese Zucker rats was much higher than that of ACBD1 and that long-chain acyl-CoA esters activated ATP-sensitive potassium channels inhibiting β-cell excitability obviously implicated ACB-containing proteins in the development of obesity and diabetes [31], [32].

Lipid-regulating function of ACBD3, however, was found so far to be restricted to cellular steroidogenesis that occurred in mitochondria [8]. Following hormone stimulation, PKA/cAMP-related signals initiated formation of a protein complex on the mitochondrial membrane, containing ACBD3, mitochondrial translocator protein, protein kinase A regulatory subunit 1a, ACBD1 and steroidogenic acute regulatory protein, and subsequently mediated cholesterol transport into mitochondria, the rate-limiting step along the pathway. According to the findings of the present study, biological functions of ACBD3 are expanded significantly into area of de novo lipogenesis to modulate fatty acid biosynthesis. Due to the embryonic lethality induced by ACBD3 gene knockout in mice and the fundamental nature of fatty acids in cell survival, growth, proliferation and death, the physiological and pathological roles undertaken by ACBD3 appear to be more important and complicated than those currently recognized in the field.

Intracellular SREBPs can be modulated by different nutritional and hormonal cues at several levels, including transcription, translation, processing (maturation) and degradation. Intracellular processing of SREBP1A and SREBP2 could be stimulated by depletion of cellular sterol content [19]. On the other hand, maturation of SREBP1A and SREBP1C could be inhibited by incubation with unsaturated fatty acids but promoted by blocking phospholipid (phosphatidylcholine) production [4], [33]. Meanwhile, expression and activation of SREBP1C is resistant to sterol deficiency but sensitive to feeding and treatment of insulin and other growth factors (as shown in Figure S5) [6]. Following receptor activation by growth factors, phosphoinositide 3-kinase (PI3K)/Akt (protein kinase B)-mediated signaling pathway seems to play a dominant role in activating SREBP1 (intensively reviewed in [34]).

SREBP processing and activity appears to also be sensitive to a variety of stressful stimuli. NMDA-mediated excitotoxicity in neuronal injury has been also linked to increased activity of SREBP1 by augmented Insig1 degradation [35]. Similarly, via depleting Insig1, both ER stress inducers and hypotonic stress inducers were found to promote proteolytic processing of SREBPs [36], [37]. Under fasting condition, activation of glucagons/cAMP/PKA signaling cascade caused direct phosphorylation at two consensus PKA recognition sites located in the ligand binding/heterodimerization domain of LXRα and concomitantly suppression of SREBP1C gene transcription [38]. Two recent studies demonstrated that Sirtuin 1, a key NAD^+^-dependent deacetylase involved in calorie restriction (CR)-mediated lifespan extension, directly deacetylate both SREBP1 and SREBP2, enhancing their ubiquitination and thus decreasing their stability [39], [40]. Together, all of these observations indicated that SREBP-regulated lipogenic pathway represents a fundamental adaptation mechanism, which is critical for cells to response to their dynamically-changed living environment.

Our results in Figure 3B and S1A clearly showed dose-dependent reduction of nuclear SREBP1 (nSREBP1) and the corresponding enhancement of membrane form (fSREBP1) at 48 hours after ACBD3 vector transfection. Moreover, it was displayed that ACBD3 physically bound with fSREBP1 but not nSREBP1 (Figure 4). Meanwhile, our results excluded possibility that ACBD3-inhibited SREBP1 maturation is due to ACBD3's disturbing effect on the physical interaction among SREBP1-SCAP-Insig1 (Figure S3). These findings strongly implicated that ACBD3, via binding with a non-N-terminal domain of fSREBP1 on the Golgi membrane (Figure 5C), blocks a process of S1P/S2P-mediated proteolytic cleavage. Considering ACBD3's putative inhibitory function on ER-to-Golgi vesicular trafficking [11], SREBP1 transfer from the ER to Golgi may be also disrupted in the presence of overexpressed ACBD3. Owing to the well-conserved proteolytic processing steps among the three SREBP family members, it is probable that ACBD3 might exert a similar impact on SREBP2. In fact, we have observed that (Figures S1 and S2) that manipulation of ACBD3 induced similar impact on SREBP2 as those of SREBP1. In addition, it is unlikely that ACBD3 directly regulates nSREBP1 post-translational modification, degradation and/or transactivation capacity because of their undetectable physical interaction (Figure 4), the ACBD3-induced upregulation of fSREBP1 (Figures 3B and S1A), and significantly different subcellular localization (cytosol vs. nucleus).

At 72 hours after transfection (Figure S1A), ACBD3 overexpressed from the lowest-dose transfection apparently boosted the fSREBP1, while those from higher-dose transfection largely downregulated the full-length protein. This discrepancy could be explained by SREBP1's auto-regulatory function and lag time between gene transcription, protein translation and degradation. We might expect to see the reduced fSREBP1 if cells were allow to overexpress ACBD3 at the lowest level for longer duration than 72 hours. This expectation was further supported by the observation that all three different levels of overexpressed ACBD3 promoted the expression of fSREBP1 at 48-hours time point.

Additionally, the different responsiveness of FASN and ACC to ACBD3 overexpression at 48 and 72 hours following transfection (Figure 6C) could also be attributed to the lag time between transcription inhibition and protein turnover of FASN and ACC. At 48 hours after transfection, pre-existing FASN and ACC seemed to contribute to the irresponsiveness even though nSREBP1 was down-regulated (Figure S1A) and transcription of the two target genes was inhibited (Figure 6A and 6B) at this point. With the prolonged ACBD3 overexpression (72 hr after transfection), significant amounts of the pre-existing proteins were degraded and there was no enough newly-synthesized proteins to compensate in the system. As a result, dose-dependent decreases of steady-state FASN and ACC were displayed under this prolonged expression condition. These findings were in accord with the results from experiments quantifying de novo palmitate synthesis (Figure 6D). Palmitate production was significantly reduced only when ACC and FASN protein levels were decreased.

Utilizing molecular and biochemical approaches (Figure 5), we found that N-terminal domain (residues 1–171) of ACBD3 played an important role in its inhibitory function on the intracellular processing and transcriptional activity of SREBP1. This observation implied that the ACB domain (residues 80–171), accounting for more-than-half portion of the deleted N-terminal sequence, might be required for ACBD3 to modulate SREBP1 activation to highest extent. Moreover, confocal imaging results showed that the ΔN mutant still localized mainly on the Golgi just like the full-length wildtype, indicating that Golgi association was not sufficient for its maximum inhibitory function. This observation, from a different perspective, supported the concept that ACB domain might involve in ACBD3-mediated SREBP1 regulation. On the other hand, deletion of C-terminal GOLD domain did not disrupt ACBD3's function to inhibit SREBP1 activity, revealing that these residues (381–526) were not required for its effect. Interestingly, unlike full-length and ΔN ACBD3, this ΔC mutant is localized more diffusely and only a small portion appeared to be located on the Golgi. Based on this finding, we still could not exclude the possibility that Golgi association contributes to ACBD3's effects on SREBP1.

As one master lipid regulator, Akt/mTORC1-regulated SREBP function is apparently fundamental for maintaining structure and energy homeostasis of cells, determining cell survival and growth. Accordingly, it is not surprising that altered SREBP expression and activity have been implicated in imbalanced lipid metabolism during the development of many dyslipidemic diseases such as obesity, diabetes, hepatic steatosis, cancer, neurodegeneration, and schizophrenia [18], [19], [41]–[44]. Thus, measures to curtail lipogenic function of SREBPs have displayed therapeutic potentials on different disorders involving imbalanced lipid metabolism. Metformin, a widely-used AMPK activator in clinics for type 2 diabetes, was reported to ameliorate fatty liver by reducing mRNA and nuclear protein of SREBP1 [45]. Through facilitating physical SCAP-Insig1 interaction, small molecule betulin inhibited SREBP maturation and subsequently attenuated target gene transcription. Betulin administration improved the lipid profiles and insulin resistance in mice fed with Western-diet and antagonized atherosclerotic lesion formation in LDLR-deficient mice [46]. Due to established roles of FASN overexpression in tumor growth and malignancy, targeting on fatty acid de novo synthesis for cancer therapy had been intensively explored. Direct FASN inhibition by its selective inhibitors was one of the most popular interventions to be tested under various in vitro and in vivo settings, and many supportive results were reported [47]–[50]. Apparently, discoveries of novel signaling pathways that regulate lipid metabolism via controlling SREBP transcription, expression and maturation are desirable from not only basic but also clinical perspectives.

The present study undoubtedly revealed that ACBD3 overexpression reduced SREBP1 maturation, transactivation ability, and ultimately, fatty acid de novo synthesis. Furthermore, with an extensively-employed cell model of SREBP regulation, ACBD3 demonstrated its potential to control cellular lipid homeostasis (Figure 2). Considering the multifaceted cellular functions of lipids, these observations suggest that ACBD3 may have many unappreciated lipid-related functions in various cells and that it would be a feasible measure to manipulate ACBD3 expression and function for modulating many SREBP1-involved physiopathological conditions.

To the best of our knowledge, the current study is the first report showing a physical and functional connection between a lipid-binding protein (ACBD3) and a master lipid regulator (SREBPs). Our findings provide evidence that ACBD3 is involved in lipid homeostasis through influencing cellular lipogenic pathway by regulating intracellular maturation of SREBP1. More studies are warranted to clarify ACBD3's biological roles in maintaining lipid homeostasis and therapeutic potentials for diseases attributed to imbalanced lipid metabolism such as obesity, diabetes, cancer and neurodegeneration.

## Materials and Methods

### Reagents and Antibodies

Dulbecco's modified Eagle's medium (DMEM, 1X) was purchased from Mediatech (Manassas, VA). NUPAGE precast SDS-PAGE gel, Fetal bovine serum (FBS), penicillin-streptomycin mixture, Alexa Fluor 594 Goat anti-mouse and Goat anti-rabbit IgGs, Lipofectamine 2000 and Neon transfection system were acquired from Invitrogen (Grand Island, NY). Polyfect transfection reagent, RNase-free DNase Set and RNeasy Mini Kit were purchased from Qiagen (Valencia, CA). Pureyield plasmid midiprep system, Luciferase assay system and dNTP were obtained from Promega (Madison WI). Quick ligation kit and SalI/NotI/BamHI/HindIII restriction enzymes were from New England Biolabs (Beverly, MA); SuperSignal West Pico chemiluminescence reagent, anti-Insig1 antibody, microscope cover glass and slide were purchased from Thermo Scientific (Waltham, MA). Phosphatase inhibitor cocktail 2 and 3, Poly-D-lysine, Cholesterol, 25-Hydroxycholesterol and (R)-Mevalonic acid sodium salt were obtained from Sigma-Aldrich (Saint Louis, MO). The following reagents were purchased from the indicated companies: Mevastatin from Cayman Chemical (Ann Arbor, MI); Delipidated FBS from Cocalico Biologicals (Reamstown, PA); PfuUltra High Fidelity DNA polymerase from Stratagene (La Jolla, CA); FractionPREP cell fractionation kit from BioVision (Mountain View, CA); anti-Myc mouse monoclonal antibody from Roche (Indianapolis, IN); anti-SREBP1 mouse monoclonal antibody from Santa Cruz (Santa Cruz, CA); primary antibodies against GAPDH, EGFR, Myc, FASN and ACC from Cell Signaling Technology (Danvers, MA); horseradish peroxidase (HRP)-conjugated goat anti-mouse and donkey anti-rabbit IgGs from Jackson ImmunoResearch (West Grove, PA); Glutathione 4B sepharose beads and HRP-conjugated anti-GST antibody from GE Healthcare (Piscataway, NJ); Anti-SREBP2 antibody from Abcam (Cambridge, MA); Anti-SCAP antibody from Bethyl Laboratories (Montgomery, TX); VECTASHIELD mounting medium with DAPI and normal goat serum from Vector Laboratories (Burlingame, CA); Deuterium oxide from Cambridge Isotope Laboratories (Andover, MA); Eagle's Minimum Essential Medium (EMEM) and pCMV-SCAP plasmid from ATCC (Manassas, VA); TrueBlot Anti-Rabbit Ig IP Beads from eBioscience (San Diego, CA); pRNAT-U6.1/Neo siRNA expression vector from GenScript (Piscataway, NJ); High Capacity cDNA Reverse Transcription Kit and Power SYBR Green PCR Master Mix from Applied Biosystems (Foster City, CA).

### Cell Lines

HEK293T [10] and HeLa (ATCC: CCL-2) cells were cultured in complete growth medium containing DMEM supplemented with 10% fetal bovine serum (FBS) and 100 U/ml penicillin-streptomycin (PS) at 37°C with 5% CO2 atmosphere in a humidified incubator. Hep G2 cells were free gift from Dr. Mitchell Lazar of University of Pennsylvania and maintained in EMEM containing 10% FBS and 100 U/ml PS at the same incubation condition as other cells.

### Generation of Constructs

Human SREBP1A (GenBank ID: U00968) cDNAs encoding the full length (1–1147) and nuclear mature form (1–490) were generated from construct pSREBP-1a (ATCC 79811) and cloned into pCMV-Myc (Clontech) between SalI and NotI sites. Plasmids encoding Myc- and GST-tagged rat full-length ACBD3 were constructed as described before [10]. cDNAs of two deletion mutants of ACBD3, ΔC (1–380) and ΔN (172–526), were made using the full-length as the template and then introduced into SalI/NotI sites of pCMV-Myc vector. Plasmids pEYFP-Golgi and FASN promoter Luciferase were obtained from Clontech (Mountain View, CA) and Addgene (Cambridge MA), respectively.

### Western Blotting

Cells were transfected with tested plasmids using Polyfect reagent (HEK293T and HeLa) or Neon Transfection System (Hep G2) according to the manufacturer protocol and then harvested at the indicated time point after transfection. Cell pellet was solubilized in buffer A (100 mM Tris [pH 7.4], 150 mM NaCl, 1% Triton X-100, 15% glycerol, phosphatase inhibitor cocktail 2 and 3, 1 mM PMSF, 25 µg/ml antipain, 50 µg/ml leupeptin, 50 µg/ml aprotinin, 25 µg/ml chymostatin, and 25 µg/ml pepstatin). Total protein of 10 µg for each sample was loaded for SDS-PAGE followed by immunoblotting with interested antibodies.

### Immunoprecipitation

GST-ACBD3-encoding vector was co-transfected with plasmid expressing Myc-tagged full-length or N-terminal of hSREBP1A into HEK293T cells. Forty-eight hours later, cells were harvested and lysed in Buffer A. Total protein of 400 µg was incubated with 40 µl of Glutathione 4B sepharose beads for overnight at 4°C. For investigating the effects of ACBD3 on the SREBP1-SCAP-Insig1 interaction, GST-ACBD3, Myc-hSREBP1 (full length) and SCAP were co-expressed in the HEK293T cells. Cell pellets were lysed in Buffer B (50 mM HEPES-KOH (pH7.4), 100 mM NaCl, 1.5 mM MgCl_2_, 0.1% (v/v) NP-40, phosphatase and protease inhibitor cocktails) [51]. Total protein of 400 µg was pre-cleared with 40 µl of TrueBlot Anti-rabbit IP beads for 1 hr at 4°C and then incubated with 4 µg of anti-SCAP antibody for overnight at 4°C followed by 40 µl of TrueBlot Anti-rabbit IP beads for 1 hr at 4°C. Then, beads were washed three times with the corresponding lysis buffer, and bound proteins were eluted with 2× SDS-loading sample buffer by boiling for 10 min. Samples were separated by SDS-PAGE and analyzed by immunoblotting. Total cell lysate (10 µg protein) was loaded as input.

### Immunofluorescence Staining and Confocal Microscopy

HEK293T cells were plated onto microscope cover glass pre-treated with poly-D-lysine (0.1 mg/ml) for overnight at room temperature. After recovery for 24 hr, cells were transfected with pEYFP-Golgi and vectors encoding Myc-tagged full-length, ΔC or ΔN ACBD3 using Polyfect. Forty hours after transfection, cells were washed two times with cold 1× PBS and then fixed with cold 4% paraformaldehyde on ice for 20 min. Following washing with PBS thrice, cells were permeabilized and blocked in Buffer C (0.1% Triton X-100, 1% BSA and 2% normal goat serum in PBS) on ice for 1 hour. Cells were subsequently labeled with anti-Myc antibody (Roche, 1∶2000) diluted in Buffer B for overnight at 4°C and then with Alexa Fluor 594-conjugated goat anti-mouse IgG antibody (1∶2000) for 1 hour at room temperature in the dark. For studying the effects of ACBD3 on the localization of endogenous SCAP and Insig1, primary antibodies against Myc (Cell Signaling, 1∶400), SCAP (1∶200), Insig1 (1∶200) and Alexa Fluor 594-conjugated goat anti-rabbit secondary antibody (1∶1000) were employed. After washing, the cover glass was mounted on the microscope slide with DAPI-containing mounting medium. Confocal fluorescent images were obtained by a Leica TCS SP5 II (Leica Microsystems, Wetzlar, Germany) scan head mounted on a Leica DMI6000B inverted-based microscope with a 63× NA 1.4 objective. Sequential excitations at 405 nm (DAPI), 513 nm (EYFP-Golgi) and 594 nm (Myc-ACBD3, SCAP and Insig1) were provided by diode, argon and helium-neon gas lasers, respectively. Blue, yellow and red fluorescent images of the same view were saved with Laser AF software. The term co-localization refers to the coincidence of yellow and red fluorescence, as measured by the confocal microscope.

### Lipid Deprivation and Cell Fractionation

HeLa cells were transfected with pCMV-Myc-ACBD3 or empty vector using Polyfect on day 1. On day 2, the growth media were replaced with either Depletion (inducing) medium (DMEM supplemented with 10% delipidated FBS, 100 U/ml PS, 50 µM Mevastatin, 50 µM Mevalonic acid sodium) or Repletion (suppressing) medium (Depletion medium supplemented with 10 µg/ml cholesterol plus 1 µg/ml 25-hydroxycholesterol). Cells were incubated at 37°C for 20 hr followed by cell fractionation to extract cellular membrane and nuclear fractions using FractionPREP kit according to the manufacturer protocol. Aliquots of the nuclear extracts (9 µg of protein) and membranes (9 µg of protein) were subjected to SDS-PAGE/immunoblot analysis.

### Luciferase Assay

HEK293T cells were seeded onto 12-well plates and cultured for 24 hr before transfection. Plasmid encoding FASN promoter luciferase was co-transfected into the cells with Myc-ACBD3 in the presence or absence of vector expressing Myc-hSREBP1A. After indicated time period, cells were rinsed with 1× PBS and harvested. Luciferase assays were subsequently carried out according to the manufacturer's protocol with Luciferase assay kit, and luciferase activity was quantified by using Synergy 4 Multi-mode Microplate Reader (BioTek Instruments, Winooski, VT). We did consider using CMV *Renilla* luciferase as the control vector to normalize transfection efficiency. All of our preliminary experiments showed that co-expression with or without *Renilla* luciferase had no effect on the degree of ACBD3-initiated inhibition onto FASN promoter, which means that transfection efficiency was not an issue for our experiment. Therefore, we did not co-transfect *Renilla* control vector in the formal experiments. All experiments were performed in triplicate.

### Knockdown of Endogenous ACBD3

Two short hairpin RNA-expressing vectors for knocking down endogenous human ACBD3 were generated using pRNAT-U6.1 (Neo) as the template. Inserts containing sense-loop-antisense sequences were cloned into the template between BamHI and HindIII sites. The two sense fragments are 5′-CCCAGCTCATAGGTGTTCATA-3′ and 5′-GCAAAGCATTTCATCCAACTT -3′ and specifically target parts of 3′-UTR and coding region of human ACBD3 transcripts, respectively. Cells were transfected with the mixture of the two plasmids (1∶1) or the control empty using Lipofectamine 2000 (HEK293T) or Neon Transfection System (Hep G2) according to the manufacturer protocol. Cells were harvested 72 hr after transfection, and then lysed for immunoblotting to determine protein expression.

### RNA Isolation and Real-time qPCR

Total RNA was isolated from HEK293T cells overexpressing Myc-ACBD3 for indicated time period using RNeasy Mini Kit according to the manufacturer's protocol. cDNAs were synthesized from 4 µg of total RNA using High Capacity cDNA Reverse Transcription Kit according to the manufacturer's manual. Ten nanogram of the resulting cDNA was subsequently mixed with Power SYBR Green PCR Master Mix and various sets of gene-specific primers and then subjected to RT-PCR quantification using the ABI 7300 Real Time PCR System (Applied Biosystems). The sequences of the primers used were as follows: FASN (forward), 5′-CGCGTGGCCGGCTACTCCTAC-3′; FASN (reverse), 5′-CGGCTGCCACACGCTCCTCT- 3′; ACC2 (forward), 5′- GGTGCTTATTGCCAACAACGGGAT-3′; ACC2 (reverse), 5′-TCTTGATGTACTCTGCGTTGGCCT-3′; GAPDH (forward), 5′- AGAAGGCTGGGGCTC ATTTG-3′; GAPDH (reverse), 5′- AGGGGCCATCCACAGTCTTC-3′. GAPDH mRNA was used as the invariant endogenous control, and melting curve analysis was run to make sure specificity of each amplicon. All reactions were performed in triplicate. The relative amounts of the RNAs were calculated by using the comparative threshold cycle method.

### Deuterium Oxide (D2O) Labeling and Quantitation of de novo Biosynthesis of Palmitate

HEK293T or Hep G2 cells were transfected (using Polyfect reagent and Neon Transfection System, respectively) with Myc-ACBD3 or empty vectors. Twenty-four or forty-eight hours following transfection, cell medium was changed with Labeling medium (Complete growth medium supplemented with 5% D2O). After labeling for 24 hours, cells were washed with 1× PBS and harvested. Cell pellet was delipidated with chloroform∶methanol (2∶1 v∶v) following the addition of heptadecanoate (17:0) as an internal standard. The lower chloroform fraction containing extracted lipids was collected following the addition of PBS (0.75 volume). The lipid fraction was then dried under nitrogen and saponified with methanolic NaOH. Fatty acids in the saponified lipid extract were analyzed as their methyl ester derivatives [52] using gas chromatography-electron impact ionization mass spectrometry using an Agilent 7890A GC- Agilent 5975-MS system, a DB-5 MS capillary column (30 m×0.25 mm×0.25 µm). The oven temperature was initially held for 2 min at 150°C, then increased by 5°C per min to 200°C, then increased by 10°C per min to 250°C. The split ratio was 10∶1 with helium flow 2 ml per min. The inlet temperature was set at 250°C and MS transfer line was set at 280°C. The ^2^H-enrichment in palmitate was determined by using selective ion monitoring under electron impact ionization of m/z 270 and 271 (M+0 and M+1), 30 ms dwell time per ion. Palmitate abundance was normalized to the heptadecanoate (17:0) internal standard (m/z 284) and the palmitate concentration determined by interpolation from a standard curve. The rate of lipid synthesis was determined as the percent contribution of newly-synthesized palmitate, using the equation: % newly-synthesized palmitate = [total ^2^H-labeling palmitate/(^2^H-labeling media×n)]×100, wherein n is the number of exchangeable hydrogens, assumed to equal 22 [53]. The absolute amount of de novo synthesized palmitate was determined by multiplying the % newly-synthesized palmitate by the concentration of palmitate. The final value was normalized against total protein amount (mg) of each sample for comparison among different groups [54].

### Data Analysis

All quantitative data were present as mean ± S.E. if they were derived from at least 3 experiments. For comparison of multiple groups, the data were analyzed by one-way analysis of variance (ANOVA) followed by a Tukey post hoc test. Student's *t*-test was employed for directly testing the difference between two sets of independent samples. If the *p* value less than 0.05, the difference was defined as significant. All statistical analyses were performed using GraphPad Prism (GraphPad Software, San Diego, CA).

## Supporting Information

Figure S1 **ACBD3 does not affect expression of SCAP and Insig1.** For investigating impacts of ACBD3 on the expression of SCAP, Insig1 and nuclear SREBP2, HEK293T (A.) and Hep G2 (B.) cells overexpressing different levels of Myc-ACBD3 for 48 or 72 hr were harvested for SDS-PAGE/Western blotting analysis as described in “Methods”.
(TIF)

Figure S2 **ACBD3 knockdown up-regulates nuclear SREBP2 but has no effect on expression of SCAP and Insig1.** HEK293T (A.) or Hep G2 (B.) cells were transfected with control or ACBD3-shRNA vectors and harvested 72 hr after transfection for SDS-PAGE/Western blotting analysis as described in “Methods”.
(TIF)

Figure S3 **ACBD3 does not affect physical SREBP1-SCAP-Insig1 interaction.** HEK293T cells co-transfected with plasmids for GST-ACBD3, Myc-hSREBP1 full-length and SCAP were harvested 48 or 72 hours after transfection (A.). Co-immunoprecipitation assay was performed using anti-SCAP antibody as described in “Methods” to evaluate the three-party interaction among SREBP1, SCAP and Insig1. Quantitative analysis (B.) of co-precipitated Myc-hSREBP1A (full length) in Figure S3A was conducted to testify that higher amount of co-IPed SREBP1A protein in GST-ACBD3 group is completely due to the higher expression level (input) of the protein. In order to evaluate cellular localization of SCAP and Insig1, HEK293T cells overexpressing Myc-ACBD3 for 48 hr were subjected to immunofluorescence staining and confocal imaging analysis as described in “Methods” (C.).
(TIF)

Figure S4 **ACBD3 inhibits de novo palmitate biosynthesis in Hep G2 cells.** For quantifying palmitate de novo synthesis, growth medium for Hep G2 cells was replaced with one containing 5% D2O (diluted in the complete medium) at 24 hr or 48 hr after transfection with Myc-ACBD3. Following 24-hours incubation, cells were harvested and lipids were extracted and determined as described in “Methods”.
(TIF)

Figure S5 **Insulin does not abolish ACBD3-inhibited SREBP1 maturation in HEK293T cells.** HEK293T cells were transfected with Myc-ACBD3 for 48 hr, and insulin (100 nM) was added into growth medium 30 min or 6 hr before harvest. Cell lysates were subjected to SDS-PAGE/Western blotting analysis as described in “Methods”.
(TIF)
