# Supplementary figures and images for: Maturation and Activity of Sterol Regulatory Element Binding Protein 1 Is Inhibited by Acyl-CoA Binding Domain Containing 3
Source: PLoS One. 2012 Nov 14;7(11):e49906. doi: 10.1371/journal.pone.0049906 (PMC3498211; doi:10.1371/journal.pone.0049906)

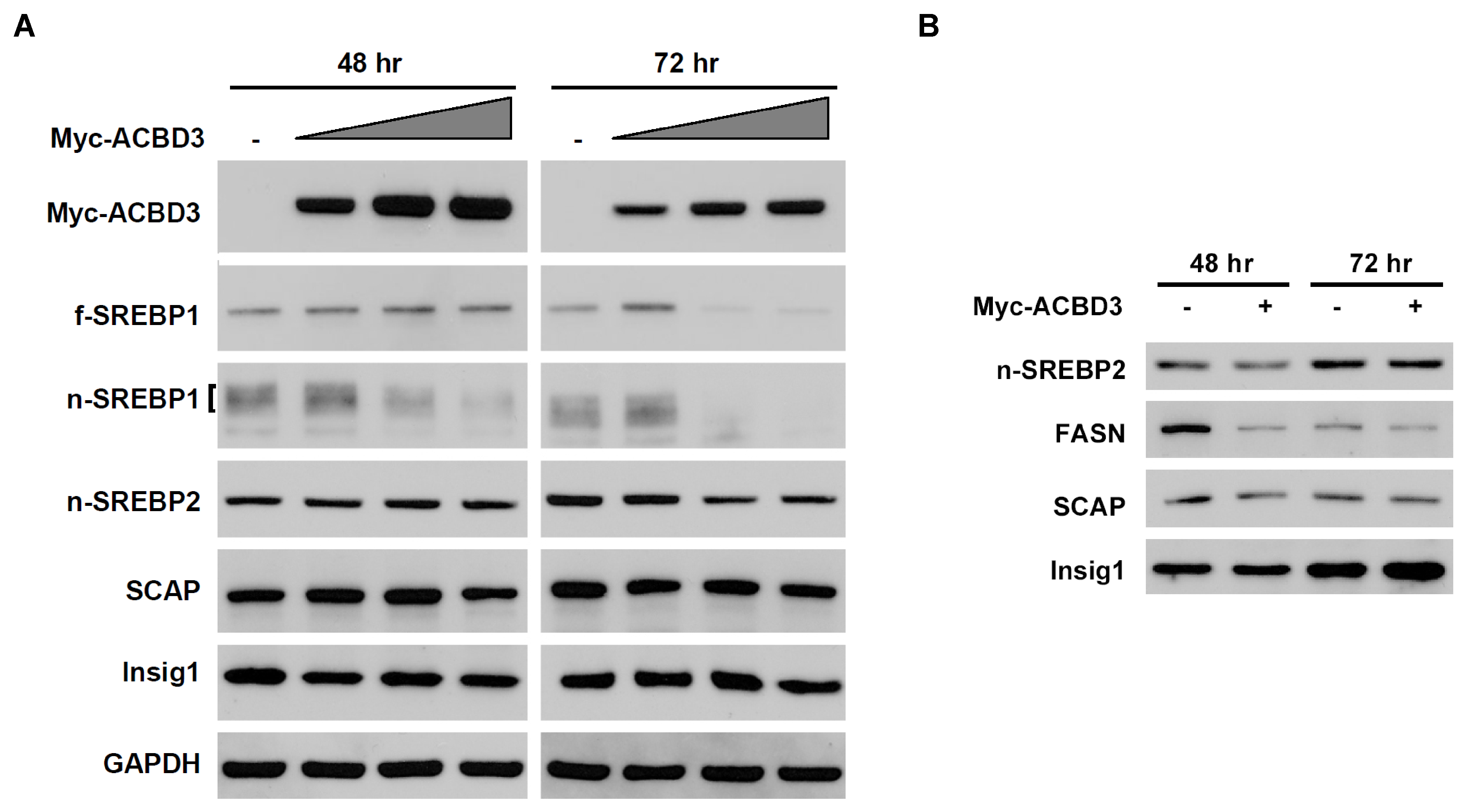

Supplement: Figure S1 — ACBD3 does not affect expression of SCAP and Insig1. For investigating impacts of ACBD3 on the expression of SCAP, Insig1 and nuclear SREBP2, HEK293T (A.) and Hep G2 (B.) cells overexpressing different levels of Myc-ACBD3 for 48 or 72 hr were harvested for SDS-PAGE/Western blotting analysis as described in “Methods”. (TIF) [file pone.0049906.s001.tif]

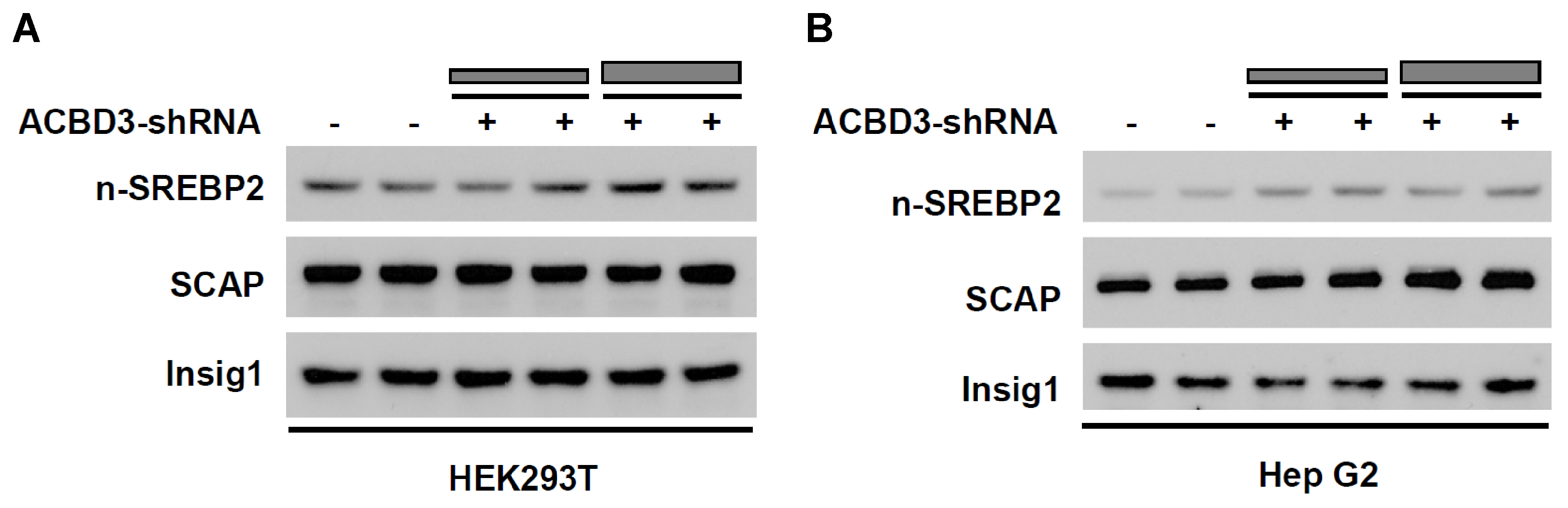

Supplement: Figure S2 — ACBD3 knockdown up-regulates nuclear SREBP2 but has no effect on expression of SCAP and Insig1. HEK293T (A.) or Hep G2 (B.) cells were transfected with control or ACBD3-shRNA vectors and harvested 72 hr after transfection for SDS-PAGE/Western blotting analysis as described in “Methods”. (TIF) [file pone.0049906.s002.tif]

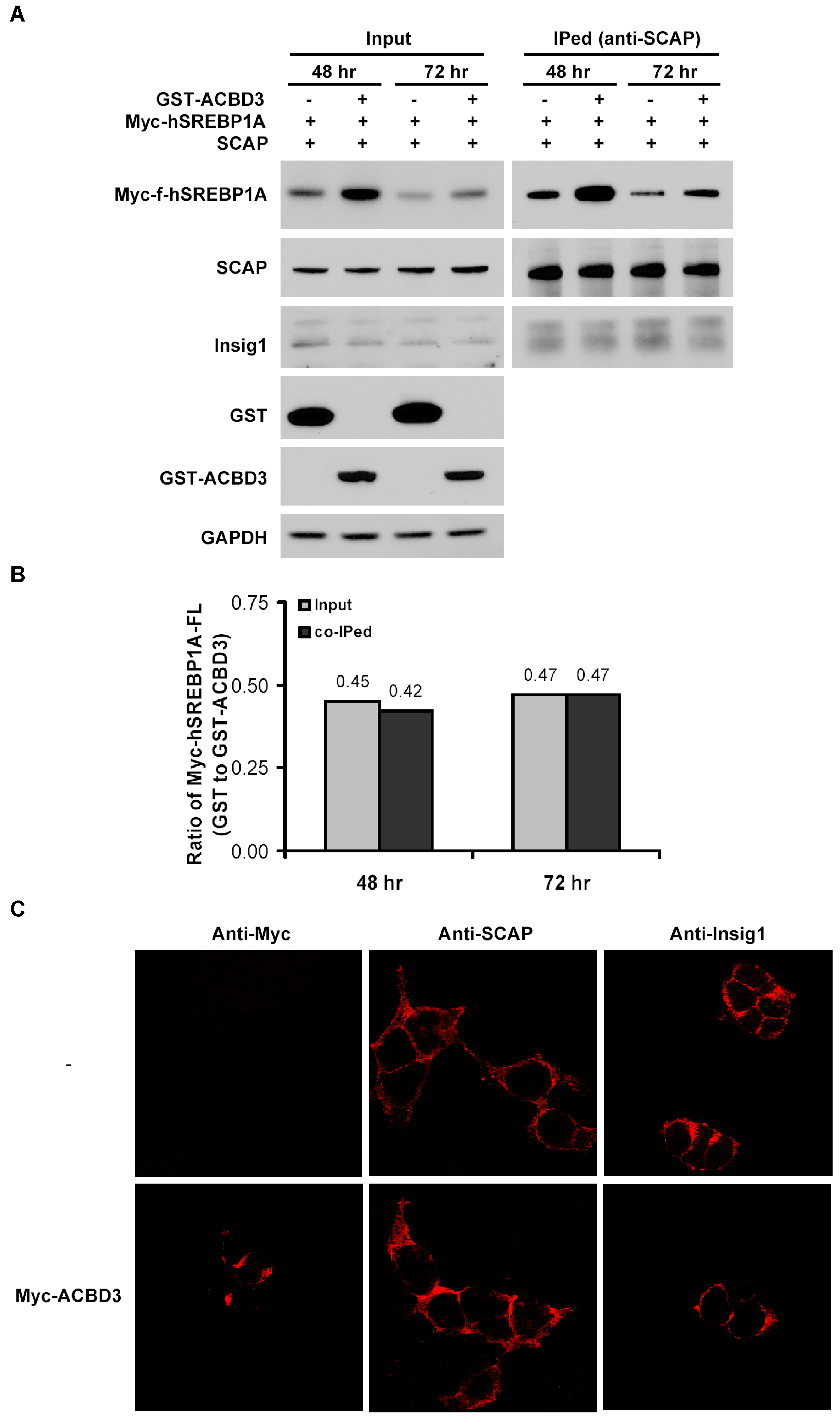

Supplement: Figure S3 — ACBD3 does not affect physical SREBP1-SCAP-Insig1 interaction. HEK293T cells co-transfected with plasmids for GST-ACBD3, Myc-hSREBP1 full-length and SCAP were harvested 48 or 72 hours after transfection (A.). Co-immunoprecipitation assay was performed using anti-SCAP antibody as described in “Methods” to evaluate the three-party interaction among SREBP1, SCAP and Insig1. Quantitative analysis (B.) of co-precipitated Myc-hSREBP1A (full length) in Figure S3A was conducted to testify that higher amount of co-IPed SREBP1A protein in GST-ACBD3 group is completely due to the higher expression level (input) of the protein. In order to evaluate cellular localization of SCAP and Insig1, HEK293T cells overexpressing Myc-ACBD3 for 48 hr were subjected to immunofluorescence staining and confocal imaging analysis as described in “Methods” (C.). (TIF) [file pone.0049906.s003.tif]

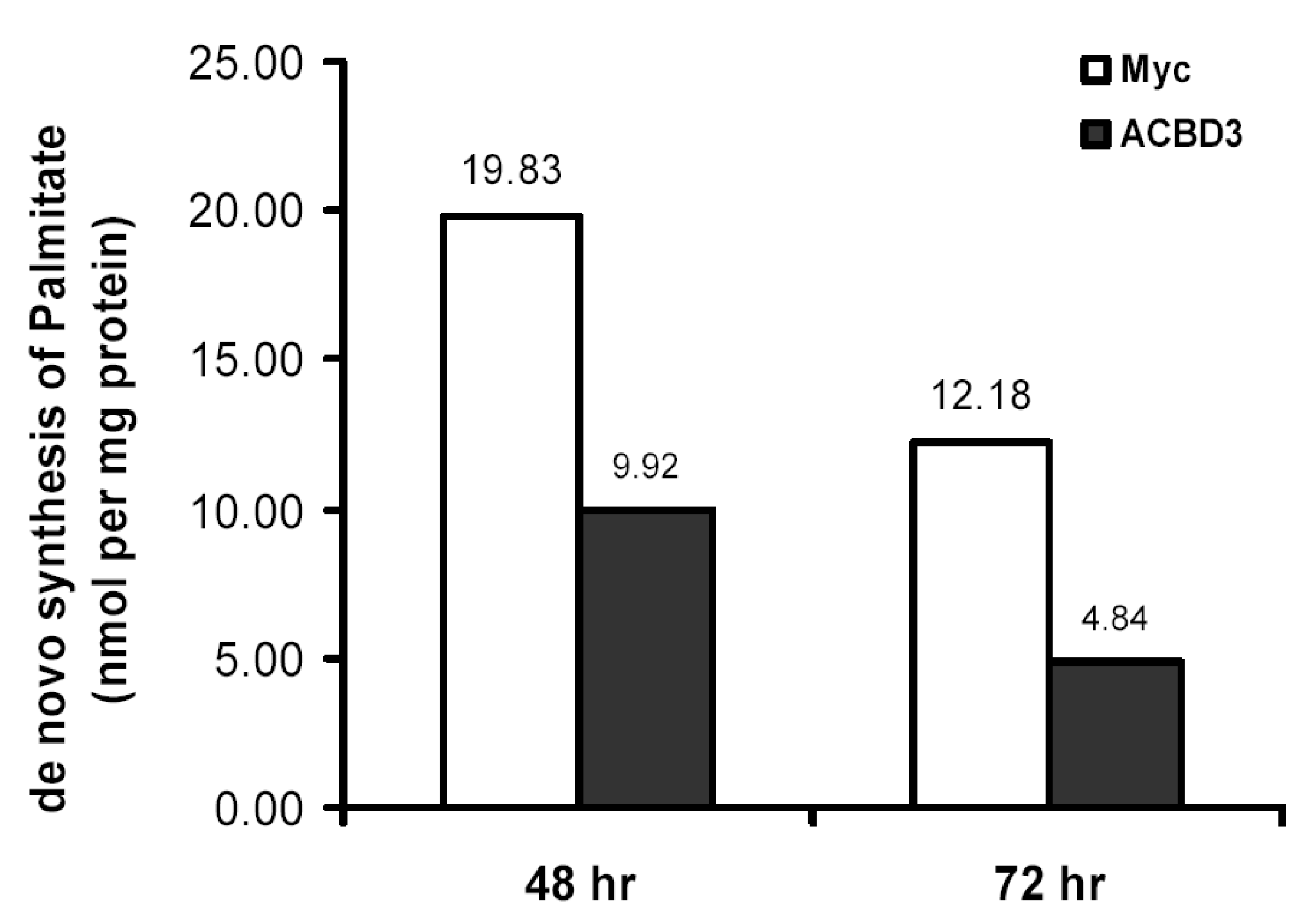

Supplement: Figure S4 — ACBD3 inhibits de novo palmitate biosynthesis in Hep G2 cells. For quantifying palmitate de novo synthesis, growth medium for Hep G2 cells was replaced with one containing 5% D2O (diluted in the complete medium) at 24 hr or 48 hr after transfection with Myc-ACBD3. Following 24-hours incubation, cells were harvested and lipids were extracted and determined as described in “Methods”. (TIF) [file pone.0049906.s004.tif]

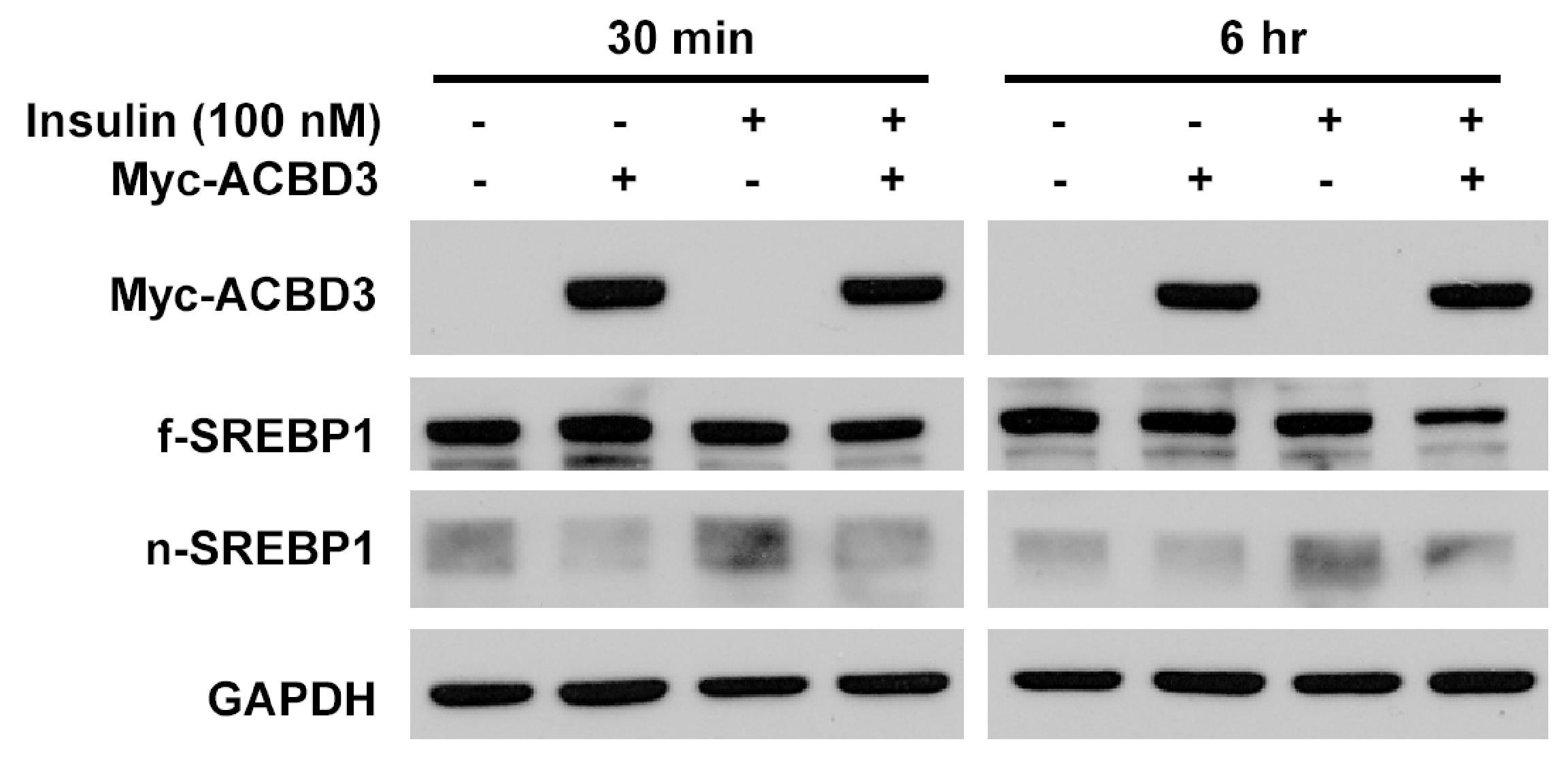

Supplement: Figure S5 — Insulin does not abolish ACBD3-inhibited SREBP1 maturation in HEK293T cells. HEK293T cells were transfected with Myc-ACBD3 for 48 hr, and insulin (100 nM) was added into growth medium 30 min or 6 hr before harvest. Cell lysates were subjected to SDS-PAGE/Western blotting analysis as described in “Methods”. (TIF) [file pone.0049906.s005.tif]
